# Characterization of *tmexCD-toprJ*-harboring plasmid in *Klebsiella pneumoniae*: insights into the molecular epidemiology of tigecycline resistance

**DOI:** 10.1128/spectrum.00689-26

**Published:** 2026-08-21

**Authors:** Yanjun Liu, Zhiqian Wang, Zijuan Jian, Peilin Liu, Qun Yan, Wenen Liu

**Affiliations:** 1 Department of Clinical Laboratory, The Central Hospital of Yongzhou, Yongzhou Clinical College, University of South China, Yongzhou, Hunan, China; 2 Department of Clinical Laboratory, Xiangya Hospital,Central South University, Changsha, Hunan, China; 3 Department of Clinical Laboratory, Nanjing Drum Tower Hospital, The Affiliated Hospital of Nanjing University Medical School, Nanjing, Jiangsu, China; 4 National Clinical Research Center for Geriatric Disorders, Xiangya Hospital, Changsha, Hunan, China; Universita degli Studi dell'Insubria, Varese, Italy

**Keywords:** carbapenem-resistant *Klebsiella pneumoniae*, tigecycline, resistance mechanisms, *tmexCD-toprJ*

## Abstract

Tigecycline-non-susceptible *Klebsiella pneumoniae* (TNSKP) is a significant global public health threat. The molecular epidemiological characteristics of the novel resistance mechanisms mediated by the mobile resistance-nodulation-cell division-type efflux pump gene cluster *tmexCD-toprJ* remain incompletely understood. Our study aims to investigate the epidemiological characteristics and genetic traits of *tmexCD-toprJ* in TNSKP. We collected 980 KP isolates from a tertiary teaching hospital between 2020 and 2021, screening for TNSKP strains by microbroth dilution. Whole-genome sequencing was used to analyze the molecular epidemiological characteristics and resistance mechanisms of TNSKP and to resolve the genetic structure of the plasmid carrying *tmexCD-toprJ*. We identified 25 TNSKP strains, including 17 carbapenem-resistant *Klebsiella pneumoniae* (CRKP) and 8 non-CRKP strains. The tigecycline MIC values were 4 µg/mL (14 strains), 8 µg/mL (nine strains), and 16 µg/mL (two strains). Multiple resistance mechanisms were detected, including *AcrR*, *RamR*, *tet*(A) mutations, and *tmexCD-toprJ*. The *tmexCD-toprJ* gene cluster was found in three strains: ST334 CRKP and ST39 CRKP (both carrying *tmexCD2-toprJ2*) and ST656 CRKP (carrying *tmexCD1-toprJ1*). ST39 CRKP also harbored *bla*_NDM-1_, *tmexCD2-toprJ2*, and *iuc3* on IncFIB(K), IncU, and IncFII(K) plasmids, with a complete conjugation element. Our research identified multiple complex tigecycline resistance mechanisms in *K. pneumoniae*, including new ST types carrying *tmexCD2-toprJ2*. A fusion plasmid harboring virulence genes, carbapenem resistance genes, and tigecycline resistance genes was discovered. Continuous molecular epidemiological monitoring plays a crucial role in elucidating the mechanisms of tigecycline resistance.

**IMPORTANCE:**

Tigecycline-non-susceptible *Klebsiella pneumoniae* (TNSKP) poses a significant global public health threat. The molecular epidemiological characteristics of the novel resistance mechanisms mediated by the mobile resistance-nodulation-cell division-type efflux pump gene cluster tmexCD-toprJ remain incompletely understood. Our study aims to investigate the epidemiological characteristics and genetic traits of tmexCD-toprJ cluster in TNSKP. This study has revealed the coexistence of multiple mechanisms contributing to tigecycline resistance. In clinical settings, we have identified various types of the tmexCD-toprJ gene cluster, among which two ST types (ST334 and ST39) carrying the tmexCD2-toprJ2 gene cluster are reported for the first time. Additionally, a fusion plasmid containing both the tmexCD2-toprJ2 gene cluster and carbapenemase resistance gene blaNDM-1 as well as virulence gene iuc3 has been detected. These findings highlight that molecular epidemiological monitoring provides important evidence for bacterial resistance and evolution. Continuous monitoring and controlling the spread of resistance from a One Health perspective are meaningful.

## INTRODUCTION

*Klebsiella pneumoniae* is a common gram-negative opportunistic pathogen in clinical settings and an important cause of nosocomial infections. It can cause pneumonia, bacteremia, and urinary tract infections, posing a serious threat to patients’ lives (1). With the widespread use of antimicrobial agents, the drug resistance of KP has become increasingly severe. In China, the resistance rate of this bacterium to third-generation cephalosporins is as high as 42.9%, and the resistance rate to carbapenems is around 27%, leading to a dilemma in clinical anti-infection treatment where “no effective drugs are available.” The mortality rate of bloodstream infections caused by carbapenem-resistant *Klebsiella pneumoniae* (CRKP) is over 20% (2).

Faced with the high incidence and mortality of CRKP, tigecycline is considered one of the few options other than polymyxins and ceftazidime/avibactam, playing an important role in both monotherapy and combination therapy. However, China Antimicrobial Surveillance Network (CHINET) data (3) shows that the resistance rate of tigecycline to *Klebsiella pneumonia* is 4.6%, and the resistance mechanisms are becoming increasingly complex. Tigecycline, as a third-generation tetracycline, is strictly speaking a novel glycylcycline antibiotic derived from tetracyclines, with an added 9-t-butyl-glycylamide side chain structure to the central framework of minocycline (4). After entering bacteria, tigecycline reversibly binds to the 16S rRNA in the 30S subunit of the ribosome, blocking the entry of tRNA into the A site, thereby inhibiting protein transcription and translation process. The currently known resistance mechanisms to tigecycline are mainly chromosomally mediated, including overexpression of active efflux pumps, mutations in the *plsC* gene that alter cell membrane permeability, and mutations in the *rpsJ* gene that reduce the affinity between tigecycline and the ribosome (5). In recent years, resistance mechanisms mediated by the plasmid-borne *tet*(X) gene (6, 7) and a novel mobile resistance-nodulation-cell division (RND) efflux pump (8) gene cluster, *tmexCD-toprJ*, have been identified. *tmexCD-toprJ* can be horizontally transferred via mobile genetic elements such as plasmids. It has already spread to animals and the environment, posing a serious threat to public health (9, 10).

Strains of *Klebsiella pneumoniae* positive for *tmexCD1-toprJ1* are more commonly found in China and Vietnam (11). *tmexCD1-toprJ1* was first discovered in *K. pneumoniae* isolated from chicken feces. Subsequently, *tmexCD2-toprJ2* was isolated from clinical samples, *tmexCD3-toprJ3* was isolated from pig feces, and *tmexCD4-toprJ4* was found in *Klebsiella quasipneumoniae* and *Enterobacter roggenkampii* isolated from chicken and environmental samples (12). The horizontal transfer mechanisms of *tmexCD-toprJ* that have been discovered so far mainly include the following four types (13). Plasmids: Hybrid plasmids are likely the main carriers of the *tmexCD1-toprJ1* gene in *tmexCD1-toprJ1*-positive *K. pneumoniae*, especially IncFIB(Mar)/IncHI1B. Insertion sequences (ISs): Insertion sequences represented by IS26 can promote the horizontal spread of *tmexCD-toprJ* genes. Transposons: Tn5393 is one of the key mobile elements for the movement of the *tmexCD1-toprJ1* gene. Integrative conjugative elements (ICE): The SXT/R391 ICE family is the most extensively studied family of integrative conjugative elements and is widely distributed among Enterobacteriaceae bacteria.

Although previous studies have revealed the existence and spread of *tmexCD-toprJ*, its specific epidemiological characteristics, genetic background, and resistance-enhancing mechanisms in clinical strains still need further exploration. This study aims to systematically analyze the molecular epidemiological characteristics and plasmid genetic environment of *tmexCD-toprJ* in clinically isolated KP through high-throughput sequencing technology, providing a theoretical basis for curbing the spread of resistance genes and optimizing clinical treatment strategies.

## MATERIALS AND METHODS

### *Klebsiella pneumoniae* strains utilized in this study, antimicrobial susceptibility test

Non-repetitive *Klebsiella pneumoniae* strains from 2020 to 2021 were collected from a teaching hospital in China. The susceptibility to antimicrobials was determined using a VITEK-2 compact system (bioMérieux). The results were interpreted according to Clinical and Laboratory Standards Institute guidelines (14). Tigecycline and colistin drug susceptibility tests were detected using the microbroth dilution technique. Tigecycline-non-susceptible *Klebsiella pneumoniae* (TNSKP) was identified based on MICs >2 mg/L according to the criteria of the US FDA. Colistin susceptibility results adhere to European Committee on Antimicrobial Susceptibility Testing guidelines (accessible at http://www.eucast.org/clinical-breakpoints/) (15).

### Efflux pump inhibition assay

Efux pump inhibitors (EPIs), carbonyl cyanide 3-chlorophenylhydrazone (CCCP, Sigma, St. Louis, Missouri, USA), were selected to evaluate the activity of efflux pumps. CCCP was prepared at a final concentration of 25 µg/mL using sterile distilled water. The MIC of tigecycline for bacteria was determined both with and without EPIs using the microdilution broth method. The efflux pump inhibitory phenotype was considered positive when the MIC of a certain strain decreased by more than or equal to fourfold in the presence of an efflux pump inhibitor (16).

### Whole-genome sequencing, bioinformatics analysis of population structure, drug-resistant genes, virulence genes, mutations, and other molecular characteristics

We utilized the Illumina NovaSeq 6000 platform for short-read sequencing to analyze the molecular characteristics of bacterial strains, including population structure, virulence genes, antibiotic resistance genes (ARGs), and mechanisms of tigecycline resistance. The optimized next-generation sequencing reads were assembled using SPAdes with multiple k-mer parameters. Genome assemblies were analyzed using Kleborate to determine taxonomic assignment, sequence type (ST), K locus (KL), O antigen (LPS) loci, and virulence loci genotypes (https://github.com/katholt/Kleborate). The PathogenWatch platform was also utilized to complement the ST assignment. Antimicrobial resistance genes were identified with ResFinder (17), and plasmid replicon types were detected using PlasmidFinder v2.1 (18).

The resistance genes *rpsJ*, *tet*(A), *tet*(X), and *tmexCD-toprJ* of the strains were analyzed using ResFinder 4.1 (dtu.dk). The nucleotide sequences and the deduced protein sequences were analyzed using DNAMAN software V8.0 (Lynnon Biosoft, USA) and compared with the reference sequences of *Klebsiella pneumoniae* MGH78578 (accession numbers: NC_009648; CP000647) to analyze the mutations in *acrR* and *ramA*.

### The definition of carbapenem-resistant hypervirulent *Klebsiella pneumoniae*

Hypervirulent strains (HvKp) were defined by the presence of virulence-associated loci (*rmpA*, *rmpA2*, *iroB*, and *iucA*) (19, 20). Carbapenem resistance was confirmed by detection of carbapenemase genes (*bla*_KPC_, *bla*_NDM_, and *bla*_OXA-48_). Strains that possess both carbapenem resistance genes and virulence genes are defined as carbapenem-resistant hypervirulent *Klebsiella pneumoniae* (CR-HvKp) (21, 22).

### The genetic structure of *tmexCD-toprJ*-bearing plasmids was analyzed using third-generation sequencing technology

Whole-genome sequencing of the three *tmexCD-toprJ* positive strains was performed using a hybrid assembly strategy incorporating long-read sequencing data generated by Oxford Nanopore Technologies (UK). This yielded three fully closed genomes. The third-generation sequencing data were assembled using Flye 2.9.2 (23), and the genome was annotated using RAST (24). MOB-suite (25) was used to predict the conjugative transfer potential. Linear alignment of the 30–40 kb regions flanking the *tmexCD-toprJ* gene cluster on the plasmids of the three strains was performed using Easyfig (26). Circular maps of plasmids were generated using Proksee. Furthermore, Easyfig was also used to conduct a linear alignment of the genetic environment of key genes located on the fused plasmid of strain KP14.

### Conjugation experiments with plasmids in *tmexCD-toprJ*-positive strains

Conjugation experiments were performed using KP6, KP14, and KP21 as donor strains and J53 as the recipient strain, respectively. After culturing the donor and recipient bacteria separately, they were mixed at a 1:1 ratio, centrifuged, and the concentrated bacterial suspension was added to an MH agar plate with a 0.22 μm filter membrane. The plate was incubated at 35°C for 20 h to complete the conjugation. Subsequently, the colonies were scraped off and serially diluted, then spread on sodium azide single-antibiotic and double-antibiotic selection plates. After incubation at 35°C for 18–48 h, the conjugation efficiency was calculated by the ratio of colony counts on the double-antibiotic to single-antibiotic plates.

## RESULTS

### Antimicrobial susceptibility test results

**Fig 1:**
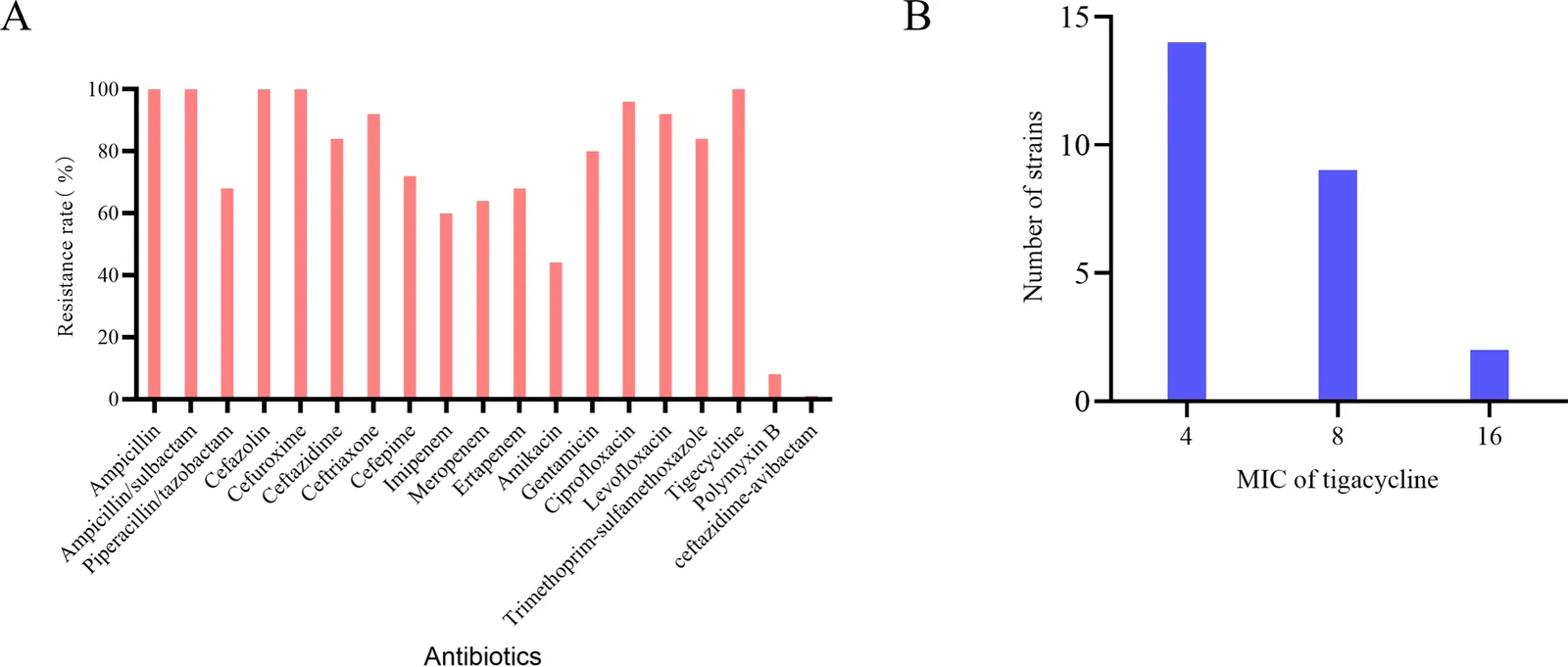
Sensitivity results of strains to antimicrobial susceptibility. (A) Susceptibility of tigecycline-non-susceptible strains to common antimicrobial drugs. (B) MIC(μg/mL) distribution of strains against tigecycline.

Among the 980 non-duplicate *Klebsiella pneumoniae* strains clinically isolated in 2020–2021, we detected 25 tigecycline-non-susceptible KP isolates. These comprised 17 CRKP and 8 non-CRKP strains. All 25 KP strains exhibited high resistance to common antimicrobial agents: ceftazidime (84%, 21/25), imipenem (60%, 15/25), levofloxacin (92%, 23/25), and amikacin (44%, 11/25). The bacteria retained high susceptibility to polymyxin, with a resistance rate of 8% (2/25). Among the 25 *Klebsiella pneumoniae* strains, 14 had a MIC of 4 μg/mL for tigecycline, 9 had a MIC of 8 μg/mL, and 2 had a MIC of 16 μg/mL. Results are summarized in Fig. 1.

### Microbiological characteristics of tigecycline-non-susceptible KP

Among the 25 TNSKP strains, the efflux pump inhibition assay showed that 64.7% (11/17) of CRKP strains and 25.0% (2/8) of non-CRKP strains could be inhibited by efflux pumps. In terms of ST types, a total of 14 ST types were detected among the 25 TNSKP strains, with 40% (10/25) being ST11, 8.0% (2/25) being ST307, 8.0% (2/25) being ST4998, and the remaining ST types each detected in one strain.

In terms of resistance genes, among the 17 CRKP strains, the predominant carbapenemase gene was *bla*_KPC-2_, accounting for 58.8%. This was followed by *bla*_NDM-1_ (11.8%, 2/17), *bla*_IMP-4_ (5.9%, 1/17), and *bla*_NDM-5_ (5.9%, 1/17). Additionally, three strains were found to carry the plasmid-mediated *tmexCD-toprJ* gene cluster associated with tigecycline resistance. One strain harbored *tmexCD1-toprJ1*, and two strains had *tmexCD2-toprJ2*. Among the 25 strains, 32.0% (8/25) carried *bla*_CTX-M-65_, and 20.0% (5/25) carried *bla*_CTX-M-15_. The detection rates of other *bla*_CTX-M_ types were as follows: *bla*_CTX-M-14_ (12%, 3/25), *bla*_CTX-M-3_ (8%, 2/25), and *bla*_CTX-M-55_ (8%, 2/25). The carriage rates of the virulence genes *rpmA*, *rmpA2, iuc*, *iro*, and *ybt* were 4% (1/25), 36.0% (9/25), 44.0% (11/25), 4.0% (1/25), and 52.0% (13/25), respectively. Results are summarized in Table 1.

**TABLE 1:** Microbiological characteristics of tigecycline-non-susceptible *Klebsiella pneumoniae*

Characteristics	CRKP	non-CRKP	Total
	(*N* = 17)	(*N* = 8)	(*N* = 25)
Tigecycline MIC			
4	12 (70.6%)	2 (25.0%)	14 (56.0%)
8	3 (17.6%)	6 (75.0%)	9 (36.0%)
16	2 (11.8%)	0 (0.0%)	2 (8.0%)
Inhibited by external pump inhibitor	11 (64.7%)	2 (25.0%)	13 (52.0%)
Sequence type			
11	10 (58.8%)	0 (0.0%)	10 (40.0%)
1,412	0 (0.0%)	1 (12.5%)	1 (4.0%)
15	1 (5.9%)	0 (0.0%)	1 (4.0%)
1,545	0 (0.0%)	1 (12.5%)	1 (4.0%)
196	1 (5.9%)	0 (0.0%)	1 (4.0%)
2,321	0 (0.0%)	1 (12.5%)	1 (4.0%)
280	0 (0.0%)	1 (12.5%)	1 (4.0%)
307	0 (0.0%)	2 (25.0%)	2 (8.0%)
334	1 (5.9%)	0 (0.0%)	1 (4.0%)
340	1 (5.9%)	0 (0.0%)	1 (4.0%)
35	0 (0.0%)	1 (12.5%)	1 (4.0%)
39	1 (5.9%)	0 (0.0%)	1 (4.0%)
4,998	1 (5.9%)	1 (12.5%)	2 (8.0%)
656	1 (5.9%)	0 (0.0%)	1 (4.0%)
Carbapenemase gene			
*bla*_KPC-2_	10 (58.8%)	0 (0.0%)	10 (40.0%)
*bla*_IMP-4_	1 (5.9%)	0 (0.0%)	1 (4.0%)
*bla*_NDM-1_	2 (11.8%)	0 (0.0%)	2 (8.0%)
*bla*_NDM-5_	1 (5.9%)	0 (0.0%)	1 (4.0%)
None	3 (17.6%)	8 (100.0%)	11 (44.0%)
ESBL gene			
*bla*_CTX-M-14_	1 (5.9%)	2 (25.0%)	3 (12.0%)
*bla*_CTX-M-15_	3 (17.6%)	2 (25.0%)	5 (20.0%)
*bla*_CTX-M-3_	1 (5.9%)	1 (12.5%)	2 (8.0%)
*bla*_CTX-M-55_	1 (5.9%)	1 (12.5%)	2 (8.0%)
*bla*_CTX-M-65_	8 (47.1%)	0 (0.0%)	8 (32.0%)
*tmexCD-toprJ*			
*tmexCD1-toprJ1*	1 (5.9%)	0 (0.0%)	1 (4.0%)
*tmexCD2-toprJ2*	2 (11.8%)	0 (0.0%)	2 (8.0%)
None	14 (82.4%)	8 (100.0%)	22 (88.0%)
Virulence gene			
*rmpA*	1 (5.9%)	0 (0.0%)	1 (4%)
*rmpA2*	9 (52.9%)	0 (0.0%)	9 (36.0%)
*iuc*	10 (58.8%)	1 (12.5%)	11 (44.0%)
*iro*	0 (0.0%)	1 (12.5%)	1 (4.0%)
*ybt*	12 (70.6%)	1 (12.5%)	13 (52.0%)

### Mechanisms of resistance of strains to tigecycline

We investigated the resistance mechanisms of 25 TNSKP strains using whole-genome second-generation sequencing. Chromosomally mediated resistance mutations in *acrR* were identified in four strains. The mutation types included p.W178L, p.Y114F, p.V165I, and p.E122K. *RamR* mutations were detected in eight strains, including p.A19V, p.T18A, p.H186N, p.Q122stop, p.A22T, p.G180D, and p.T43K. Tet-related genes were found in 11 strains, including *tet*(A). v1, *tet*(A).v2, *tet*(B).v2, *tet*(D), and *tet*R. Plasmid-mediated *tmexCD-toprJ* gene clusters were identified in three strains, including the types *tmexCD1-toprJ1* and *tmexCD2-toprJ2*. No plasmid-mediated *tet*(X) genes were detected.

Multiple strains carry multiple resistance mechanisms. Among them, strain KP6 harbors the *tmexCD2-toprJ2* gene cluster and also possesses chromosomally mediated mutations, including *acrR* p.Y114F, acrR p.V165I (frame restored), *ramR* p.H186N, and *tet*(A).v1, resulting in high-level tigecycline resistance (MIC = 16 µg/mL). Results are summarized in Table 2.

**TABLE 2:** Mechanisms of resistance of strains to tigecycline^*a*^

Isolate	Species	Tigecycline	AcrR	RamR	Tet	*tmexCD-toprJ*
		MIC (µg/mL)				
KP1	*Klebsiella pneumoniae*	4	–	–	*tet*(A).v1	–
KP2	*Klebsiella pneumoniae*	4	*acrR* p.W178L	*ramR* p.A19V	–	–
KP3	*Klebsiella pneumoniae*	4	–	–	*tet*(A).v1	–
KP4	*Klebsiella pneumoniae*	8	–	–	–	–
KP5	*Klebsiella quasipneumoniae subsp. quasipneumoniae*	8	*acrR* p.Y114F	*ramR* p.T18A	–	–
KP6	*Klebsiella quasipneumoniae subsp. similipneumoniae*	16	*acrR* p.Y114F *acrR* p.V165I - Frame restored	*ramR* p.H186N	*tet*(A).v1	*tmexCD2-toprJ2*
KP8	*Klebsiella pneumoniae*	8	–	–	–	–
KP12	*Klebsiella pneumoniae*	4	–	–	–	–
KP13	*Klebsiella pneumoniae*	8	–	–	–	–
KP14	*Klebsiella pneumoniae*	4	–	–	–	*tmexCD2-toprJ2*
KP15	*Klebsiella pneumoniae*	4	–	–	–	–
KP16	*Klebsiella pneumoniae*	4	–	–	*tet*(A).v1	–
KP17	*Klebsiella pneumoniae*	4	–	–	–	–
KP18	*Klebsiella pneumoniae*	4	–	*ramR* p.Q122stop	*tet*(A).v1	–
KP19	*Klebsiella pneumoniae*	4	–	–	*tet*(B).v2;*tet*(D);*tet*R	–
KP20	*Klebsiella pneumoniae*	4	–	*ramR* p.A22T	*tet*(A).v1	–
KP21	*Klebsiella pneumoniae*	4	*acrR* p.E122K *acrR* p.M123stop	–	–	*tmexCD1-toprJ1*
KP22	*Klebsiella pneumoniae*	4	–	–	–	–
KP23	*Klebsiella pneumoniae*	16	–	*ramR* p.Q122stop	*tet*(A).v1	–
KP24	*Klebsiella pneumoniae*	4	–	–	*tet*(A).v2	–
KP26	*Klebsiella pneumoniae*	8	–	*ramR* p.G180D	–	–
KP29	*Klebsiella pneumoniae*	8	–	–	*tet*(A).v1;*tet*(D)	–
KP34	*Klebsiella pneumoniae*	8	–	*ramR* p.T43K	*tet*(A).v1	–
KP35	*Klebsiella pneumoniae*	8	–	–	–	–
KP38	*Klebsiella pneumoniae*	8	–	–	–	–

^*a*^ –, not detected.

### Microbiological and clinical characteristics of three *tmexCD-toprJ*-positive clinical strains

We further analyzed the microbiological characteristics of the strains harboring the *tmexCD-toprJ* gene cluster. The results showed that KP6 was identified as *Klebsiella quasipneumoniae subsp. similipneumoniae*, ST334, with O5 and KL103. In addition to harboring the *tmexCD2-toprJ2* gene cluster, the strain also carries mutations in *AcrR* and *RamR*. The carbapenem resistance mechanism is attributed to the production of *bla*_NDM-1_. No virulence genes were detected.

Strain KP14 harbors the *tmexCD2-toprJ2* gene cluster, and no other tigecycline resistance mechanisms were detected. The strain is ST39, with O1/O2v2 and KL23. The carbapenem resistance mechanism is attributed to the carriage of *bla*_NDM-1_. The virulence gene *iuc3* was detected.

Strain KP21 harbors the *tmexCD1-toprJ1* gene cluster and was found to have *AcrR* mutations (acrR p.E122K and acrR p.M123 stop codon). The strain is ST656, with O102 and KL138. No virulence genes were detected. Results are summarized in Table 3.

**TABLE 3:** Microbiological characteristics of three *tmexCD-toprJ* harboring isolates^*a*^

Characteristics	KP6	KP14	KP21
Species	*Klebsiella quasipneumoniae subsp. similipneumoniae*	*Klebsiella pneumoniae*	*Klebsiella pneumoniae*
TMexD type	*tmexCD2-toprJ2*	*tmexCD2-toprJ2*	*tmexCD1-toprJ1*
AcrR	*acrR* p.Y114F *acrR* p.V165I - Frame restored	–	*acrR* p.E122K *acrR* p.M123 stop codon
RAMR	*ramR* p.H186N	–	–
ST type	ST334	ST39	ST656
O_locus	O5	O1/O2v2	OL102
K_locus	KL103	KL23	KL138
Carbapenemase gene	*bla*_NDM-1_	*bla*_NDM-1_	–
Virulence gene	–	*iuc3*	–
Tigecycline MIC	16	4	4

^*a*^ –, not detected.

### Genomic characteristics of plasmids of three *tmexCD-toprJ* harboring isolates

We further analyzed the genetic characteristics of the plasmid carrying *tmexCD-toprJ* in the strain through nanopore sequencing. Results showed that KP6 contained one chromosome and two plasmids. The *tmexCD2-toprJ2* bearing plasmid was 461,617 bp in size, with plasmid replicons IncFIB(K) (pCAV1099), IncR, and IncU. It carried multiple multidrug resistance genes, including *tmexCD2-toprJ2, aac(3)-IId*, *aadA5*, *armA*, *bla*_SFO-1_, *bla*_SHV-12_, *bla*_NDM-1_, *fosA3*, *msr(E*), *mph(E*), *qnrS1*, *sul1*, and *dfrA1*. No virulence genes were detected. Online software prediction indicated that the pKP6_tmex plasmid is conjugative, harboring four key conjugative transfer elements: oriT (origin of transfer), relaxase, T4CP (type IV coupling protein), and T4SS (type IV secretion system).

KP14 contains one chromosome and one plasmid. The *tmexCD2-toprJ2*-bearing plasmid is 531,258 bp in size, with plasmid replication types IncFIB(K), IncU, and IncFII(K), carrying resistance genes *aadA16*, *aadA5*, *bla*_OXY-2-8_, *bla*_NDM-1_, *tmexCD2-toprJ2*, *tet*(A), and *sul1*. Notably, it also contains the virulence gene *iuc3*. pKP14_tmex plasmid also possesses a complete set of conjugative elements.

KP21 contains one chromosome and three plasmids. The *tmexCD1-toprJ1* bearing plasmid is 172,903 bp in size, with plasmid replicons IncFIB(K) (pCAV1099-114) and IncHI1B (pNDM-MAR). The plasmid carries the following resistance genes: *aph(6)-Id*, *aph(3')-Ia*, *aph(3'')-Ib*, *armA*, *bla*_DHA-1_, *msr(E*), *mph(E*), *qnrB4*, *sul1*, *tmexCD1-toprJ1*, and *tet(B*). It does not contain virulence genes. None of the four conjugative elements was detected in pKP21_tmex. Results are summarized in Table 4.

172,903 bp in size, with plasmid replicons IncFIB(K) (pCAV1099-114) and IncHI1B (pNDM-MAR). The plasmid carries the following resistance genes: *aph(6)-Id*, *aph(3')-Ia*, *aph(3'')-Ib*, *armA*, *bla*_DHA-1_, *msr(E*), *mph(E*), *qnrB4*, *sul1*, *tmexCD1-toprJ1*, *tet(B*). It does not contain virulence genes. None of the four conjugative elements were detected in pKP21_tmex. Results are summarized in Table 4.

**TABLE 4:** Genomic characteristics of three *tmexCD-toprJ* bearing plasmids^*a*^

Isolate	Chromosome or plasmid/size	Plasmid type	Resistance genes	Aerobactin	Conjugal transfer
KP6	Chromosome (5,258,623 bp)	–	*bla*_OKP-B-1_ and *fosA*	–	/
	Plasmid (461,617 bp) (**pKP6_tmex)**	IncFIB(K)(pCAV1099), IncR, IncU	***tmexCD2-toprJ2*,** *aac(3)-IId*, *aadA5*, *armA*, *bla*_SFO-1_, *bla*_SHV-12_, *bla*_NDM-1_, *fosA3*, *msr(E*), *mph(E*), *qnrS1*, *sul1*, and *dfrA1*	–	oriT region (+), Relaxase (+), T4CP (+), and T4SS gene cluster (+)
	Plasmid (108,689 bp)	repB(R1701)	*qnrS1*, *sul1*, *tet(A*), and *dfrA1*	–	/
	Plasmid 4,353	Col440I	–	–	/
KP14	Chromosome (5,377,375 bp)	–	*bla*_SHV-1_*, fosA*, *OqxB*, and *OqxA*	–	/
	Plasmid (531,258 bp) (**pKP14_tmex)**	IncFIB(K), IncU, and IncFII(K)	*aadA16*, *aadA5*, *bla*_OXY-2-8_, *bla*_NDM-1_, ***tmexCD2-toprJ2***, *tet(A*), and *sul1*	*iuc3*	oriT region (+), Relaxase (+), T4CP (+), and T4SS gene cluster (+)
KP21	Chromosome (5,138,359 bp)	–	*bla*_SHV-187_	–	/
	Plasmid (172,903 bp) (**pKP21_tmex)**	IncFIB(K)(pCAV1099-114) and IncHI1B(pNDM-MAR)	*aph(6)-Id*, *aph(3')-Ia*, *aph(3'')-Ib*, *armA*, *bla*_DHA-1_, *msr(E*), *mph(E*), *qnrB4*, *sul1*, and ***tmexCD1*-*toprJ1***, *tet(B*)	–	oriT region (−), Relaxase (−), T4CP (−), and T4SS gene cluster (−)
	Plasmid (93,640 bp)	IncFII(K) and repB(R1701)	*bla*_CTX-M-15_ and *bla*_TEM-1B_	–	/
	Plasmid (86,891 bp)	IncR and IncFIA(HI1)	*aac(3)-IId*, *aadA16*, *aph(6)-Id*, *aph(3'')-Ib*, *aac(6')-Ib-cr*, *bla*_DHA-1_, *bla*_TEM-1B_, *floR*, *qnrB6*, *qnrB4*, *ARR-3*, *sul1*, *sul2*, *tet(D*), and *dfrA27*	–	/

^*a*^ /, not analyzed ; –, not detected.

### Comparative analysis of the *tmexCD-toprJ* gene clusters among the three isolates

**Fig 2:**
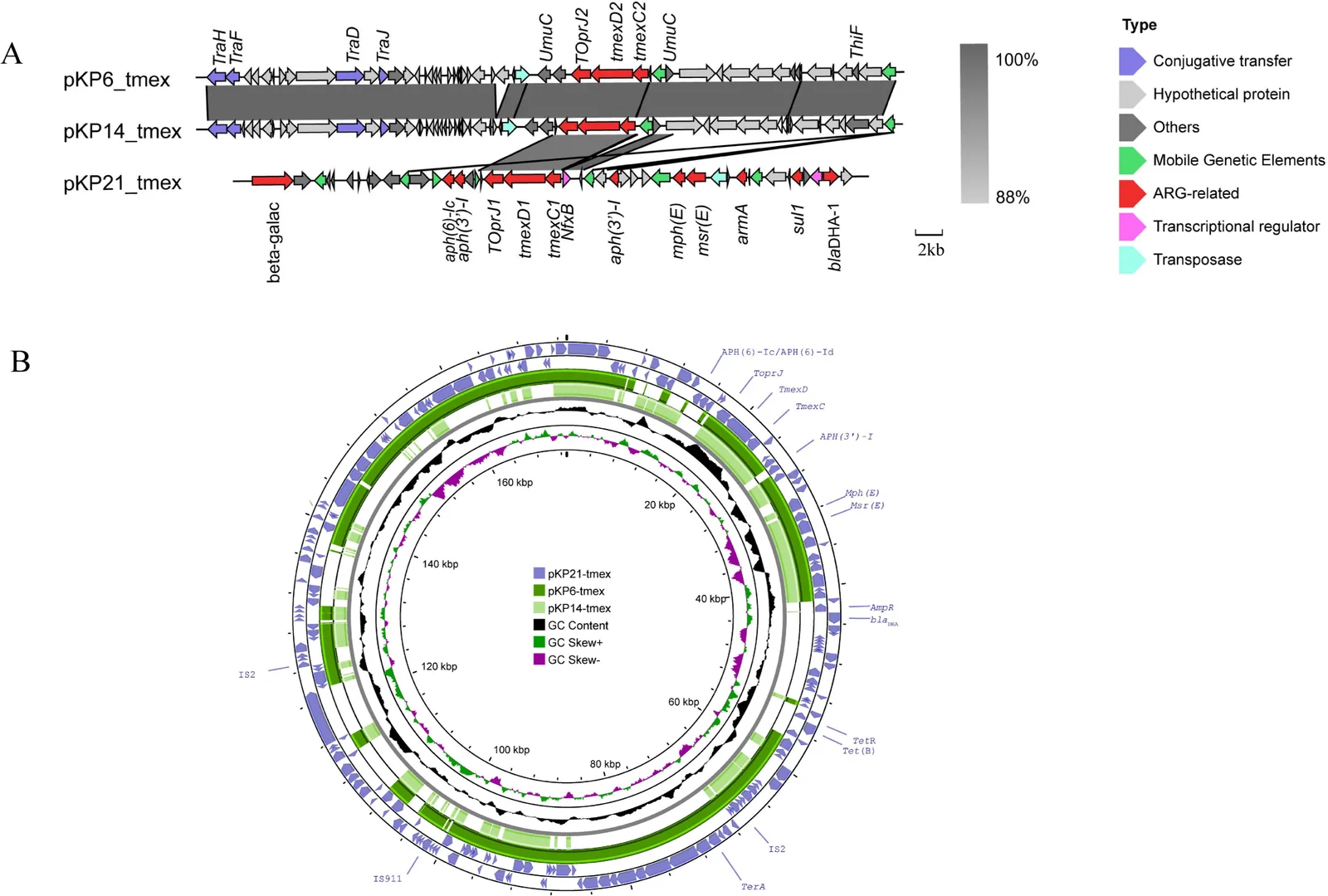
Comparison of the genetic environments of the *tmexCD-toprJ* gene cluster among the three strains. (A) Linear comparison of the 30–40 kb flanking genetic environment of the *tmexCD-toprJ* gene cluster. (B) Circular map of the genetic environment surrounding the *tmexCD-toprJ* gene cluster.

To address the transmission dynamics and evolutionary relationships of the tmexCD-toprJ gene cluster within these clinical isolates, we performed a detailed comparative analysis of the plasmid genetic backbones and flanking regions. The *tmexCD-toprJ* gene clusters in these three isolates exhibited extremely high conservation in terms of genetic composition, arrangement, and orientation. The gray shaded areas indicate that the nucleotide sequence similarity in these core regions exceeded 88%, with some regions reaching up to 100%. This suggests that the *tmexCD-toprJ* gene cluster maintains structural and functional stability across different genetic backgrounds. The upstream flanking regions of the pKP6_tmex and pKP14_tmex gene clusters showed high similarity, and both contained conjugative transfer elements (*TraH*, *TraF*, *TraD*, and *TraJ*), suggesting potential nosocomial transmission between them. In contrast, the pKP21_tmex gene cluster exhibited a more complex genetic environment. Its flanking regions were enriched with various ARGs such as aph(3')-I, *mph(E*), *msr(E*), *armA*, *sul1*, and *bla*_DHA-1_ and transposases, indicating it may represent a genomic island or a transposable unit that has integrated multiple resistance determinants. Detailed results are shown in Fig. 2A.

To comprehensively characterize the plasmid backbones and functional elements carrying the *tmexCD-toprJ* gene cluster, we generated circular maps of the representative plasmids pKP21-tmex, pKP6-tmex, and pKP14-tmex. These maps illustrate the genetic structure of the *tmexCD-toprJ* gene cluster, which spans approximately ~170 kb. The locations of various ARGs are annotated, including *aph(6)-Id*, *aph(3')-I*, *mph(E*), *msr(E*), and *bla*_DHA_. Insertion sequences such as IS2 and IS911 were found flanking the resistance genes, suggesting that these regions may have originated from exogenous horizontal gene transfer. Detailed results are shown in Fig. 2B.

Comparative genomics revealed that pKP6_tmex exhibits high homology with *Klebsiella pneumoniae* pC6364_NDM, *Klebsiella pneumoniae* pNUITM-VK2, and *Klebsiella quasipneumoniae* pNUITM-VK4 in the database. pKP14_tmex exhibits high homology with *Klebsiella pneumoniae* pNUITM-VK2, *Klebsiella quasipneumoniae* pNUITM-VK4, and *Raoultella ornithinolytica* p117885-OXY. Comparative genomics revealed that pKP21_tmex exhibits high homology with *Klebsiella oxytoca* strain p45-1, *Klebsiella pneumoniae* strain pT14441, and *Klebsiella pneumoniae* strain pTSYCC10-2 in the database. Results are summarized in Fig. S1A-C.

### Analysis of the genetic environment of the pKP14_tmex fusion plasmid

To elucidate the modular characteristics of this fusion plasmid, we further analyzed the genetic environments of key genes on the fusion plasmid of strain KP14, which simultaneously harbors the *tmexCD-oprJ* gene cluster (pKP14_tmexCD2-toprJ2), a carbapenemase gene (pKP14_NDM-1), and virulence genes (pKP14_iuc).

**Fig 3:**
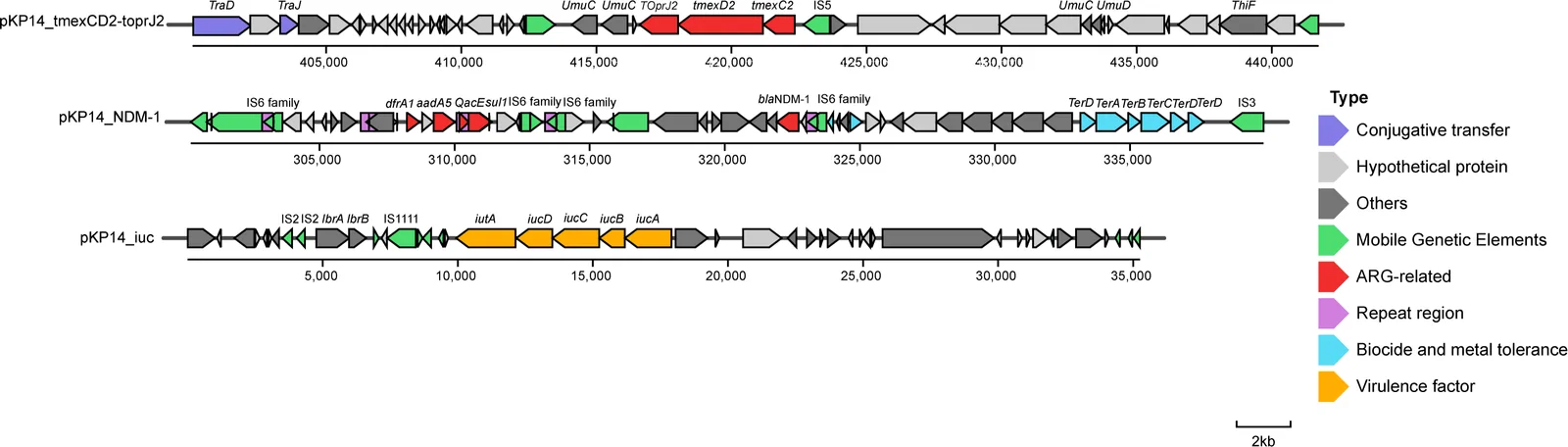
Genetic environment of key genes on the fusion plasmid (pKP14_tmex) of strain KP14. (A) Genetic structures of the region carrying the *tmexCD-toprJ* gene cluster. (B) The region carrying the carbapenemase gene *bla*_NDM-1_ (pKP14_NDM-1). (C) The region carrying the *iuc* gene cluster (pKP14_iuc).

In the pKP14_tmexCD2-toprJ2 region, the gene cluster is flanked upstream by tra genes associated with conjugative transfer and downstream by the insertion sequence IS5. Additionally, the *umuC* and *umuD* genes, which are involved in the SOS response and DNA damage repair, were identified in the flanking regions. The pKP14_NDM-1 region is associated with a complex resistance island containing *aadA5*, *dfrA1*, *qacE*, and a tellurite resistance gene cluster. Notably, an IS6 family insertion sequence was identified downstream of *bla*_NDM-1_. In the pKP14_iuc region, a complete aerobactin synthesis gene cluster (*iucABCD*) was identified, flanked by IS2 and IS1111. Detailed results are summarized in Fig. 3.

### Capacity of horizontal transfer of three *tmexCD-toprJ*-carrying plasmids

Next, we validated the conjugation transfer capability of the *tmexCD-toprJ*-carrying plasmids through conjugation experiments. The genetic structure analysis of the pKP6_tmex and pKP14_tmex plasmids revealed the presence of complete conjugative elements; however, conjugation experiments showed that they could not be successfully transferred. pKP21_tmex failed to grow on the dual-antibiotic plate, confirming its non-transferability, consistent with MOB-suite prediction results.

conjugation experiments. The genetic structure analysis of the pKP6_tmex and pKP14_tmex plasmids revealed the presence of complete conjugative elements; however, conjugation experiments showed that they could not be successfully transferred. pKP21_tmex failed to grow on the dual-antibiotic plate, confirming its non-transferability, consistent with MOB-suite prediction results.

## DISCUSSION

Our study provides a comprehensive snapshot of the molecular epidemiology and resistance mechanisms of TNSKP in a tertiary hospital in central southern China, offering a new perspective for the treatment and infection control of TNSKP.

In this study, 25 TNSKP strains exhibited rich genetic diversity, with a total of 14 STs identified, among which ST11 was the dominant type (40%, 10/25). Sixty-eight percent (17/25) of the strains were CRKP. Analysis of resistance mechanisms showed that 44.0% of the strains did not produce carbapenemase, and among the enzyme-producing strains, KPC enzyme had the highest proportion (40%, 10/25). Notably, all three strains carrying the *tmexCD-toprJ* gene belonged to CRKP. Further detection of virulence genes revealed that TNSKP strains generally carried virulence genes, which were mainly concentrated in the CRKP group, with the *iuc* gene detected in CRKP at a high rate of 58.8% (10/17). These findings confirm that *Klebsiella pneumoniae* with characteristics of tigecycline resistance, carbapenem resistance, and high virulence has already disseminated in the clinical environment, posing a severe challenge to clinical treatment and requiring high attention. Continuous molecular epidemiological surveillance is of great scientific value for grasping the evolution of *Klebsiella pneumoniae* resistance.

In terms of resistance mechanisms, the study found that the tigecycline resistance mechanisms of the strains were also diverse. For most strains, resistance was not mediated by a single mechanism but rather by the combined action of multiple mechanisms. Consistent with other studies (27), we found that most TNSKP strains had *RamR* mutations (32%, 8/25) and type 1 *tet*(A) mutations (40%, 10/25), with five strains having both *RamR* and *tet*(A) mutations. In the case of *tet*(A) mutation, the loss of *RamR* protein has a synergistic effect on tigecycline resistance in *Klebsiella pneumoniae. AcrR* mutation is also one of the mechanisms leading to tigecycline resistance. We did not detect *tet*(X), but we identified three strains carrying the plasmid-mediated *tmexCD-toprJ* gene cluster. Notably, one strain of *Klebsiella quasipneumoniae subsp. similipneumoniae* harbored four resistance mechanisms, including the acrR p.Y114F, ramR p.H186N, *tet*(A).*v1*, and *tmexCD2-toprJ2*. The combined action of these multiple mechanisms led to high-level tigecycline resistance. We speculate that the concurrent mediation at both chromosomal and plasmid levels may originate from the selective pressure of antibiotics or changes in the environment, leading to the diversification of resistance mechanisms. Meanwhile, a subset of tigecycline-non-susceptible strains in this study did not carry the canonical chromosomal mutations (*acrR* and *ramR*) or the plasmid-mediated resistance genes (*tmexCD-toprJ* and *tet*) mentioned above. We hypothesize that other mechanisms, such as mutations in global regulatory systems (*marR*, *soxR*, or *baeR*) or the presence of novel, uncharacterized plasmid-mediated genes, may also contribute to increased tigecycline resistance.

This study investigated the unique genetic characteristics of the *tmexCD-toprJ* gene cluster in three clinical isolates, revealing the localized transmission of this tigecycline resistance gene cluster. In recent years, the plasmid-encoded mobile RND-type efflux pump gene cluster *tmexCD-toprJ* has emerged as a key mechanism for tigecycline resistance in gram-negative bacteria. Since its initial identification in 2022, this gene cluster has been detected across 33 countries (28), hosts, and environmental samples worldwide (29, 30), demonstrating significant potential for widespread dissemination. Notably, distinct variants exhibit marked distribution differences: *tmexCD1-toprJ1* is widely present in animal, human, and environmental samples (9), whereas *tmexCD2-toprJ2* and *tmexCD3-toprJ3* are currently confined primarily to human sources. This distribution disparity suggests potential variations in host adaptation and transmission pathways among variants, necessitating enhanced surveillance from a One Health perspective. Among 25 clinical isolates, one was found to carry *tmexCD1-toprJ1*, and two carried *tmexCD2-toprJ2* (with ST334 and ST39 types being reported for the first time). This further confirms the diversity of this gene cluster in clinical settings and indicates that plasmid-mediated horizontal transmission (31) is a key driver of its spread. The study revealed high homology of the *tmexCD-toprJ* gene cluster between pKP6_tmex in ST334 strains and pKP14_tmex in ST39 strains. The presence of conjugative transfer elements (tra genes) upstream of the plasmids suggests they possess self-transferability, which may promote nosocomial transmission. In contrast, the *tmexCD-toprJ* gene cluster on pKP21_tmex is flanked by transposases and multiple other resistance genes, suggesting that this gene cluster may have been captured by composite transposons or genomic islands. These findings expand our understanding of the genetic background diversity among *tmexCD-toprJ*-positive strains and indicate that this gene cluster continues to spread into new clonal backgrounds, demonstrating significant evolutionary potential.

In this study, we report for the first time a fusion plasmid that integrates tigecycline resistance, carbapenem resistance, and virulence genes. Recent reports (32, 33) have documented the coexistence of *tmexCD-toprJ* with carbapenem resistance (*bla*_NDM_, *bla*_KPC_, *bla*_OXA-48_, or *bla*_IMP_), colistin resistance (*mcr*), or tigecycline resistance (*tet(X*)) genes in a single strain. For example, the co-occurrence of *mcr-1.1*, *mcr-3.40*, and *tmexCD1-toprJ1* on a hybrid IncFIB/IncHI1B plasmid in *Klebsiella pneumoniae* from pork (34), and the coexistence of plasmid-mediated *tmexCD2-toprJ2*, *bla*_IMP-4_, and *bla*_NDM-1_ in *Klebsiella quasipneumoniae* (35). These findings suggest that the integrated spread of multiple drug resistance genes and tigecycline resistance genes has become an important feature of bacterial adaptive evolution. Notably, we detected a CR-HvKp strain harboring *tmexCD-toprJ*. China is a high-prevalence area of CR-HvKp, with the ST11 clone group being the main circulating type (36). The ST39 type CR-HvKp strain identified in this study further enriches its genetic diversity. The plasmid carried by this strain (with replicons IncFIB(K), IncU, and IncFII(K)) simultaneously contains *tmexCD2-toprJ2*, *bla*_NDM-1_, virulence genes (*iuc*), and multiple other resistance genes (*aadA16*, *aadA5*, *bla*_OXY-2-8_, *tet*(A), and *sul1*).

We hypothesize that its formation likely relies on horizontal gene transfer and recombination events mediated by mobile genetic elements. Specifically, recombination mediated by insertion sequences may capture the *tmexCD-toprJ* gene cluster and virulence island into the original carbapenem-resistant plasmid, ultimately forming a superplasmid. Comparative genomic analysis revealed that the upstream and downstream regions of three key genes contain ISs: IS5 is located downstream of *tmexCD2-toprJ2*, IS6 flanks *bla*_NDM-1_, and IS1111 is adjacent to the *iuc* gene cluster. This suggests that horizontal gene transfer events mediated by these ISs may have facilitated the formation of the fusion plasmid. Furthermore, this plasmid shares high homology with certain known plasmids in the database (e.g., pNUITM-VK2), suggesting that homologous recombination may also contribute to its evolutionary process. The emergence of this fusion plasmid may be driven by the combined pressure of carbapenem and tigecycline drugs (37), and it also enhances its pathogenic potential through the synergistic expression of virulence genes, leading to the formation of the hypervirulent and highly resistant phenotype. The formation mechanism of conjugative plasmids still needs to be further investigated. In summary, the prevalence of such strains poses a severe challenge to clinical anti-infection treatment and hospital infection control. Therefore, it is urgent to strengthen molecular epidemiological surveillance, clarify the transmission routes and evolutionary dynamics of the fusion plasmid, and provide a scientific basis for curbing its spread.

### Limitations

This study has several limitations. First, it is a single-center retrospective analysis with a limited sample size, which may affect the generalizability of the findings. Second, the study focused on human clinical isolates; inclusion of environmental and animal samples would provide a more comprehensive One Health perspective on transmission dynamics. Finally, future studies will further validate the resistance function of the *tmexCD-toprJ* gene cluster directly through gene knockout and/or heterologous expression, providing more robust experimental evidence for the role of this gene cluster in tigecycline non-susceptibility.

### Conclusion

Our study has revealed the coexistence of multiple mechanisms contributing to tigecycline resistance in *Klebsiella pneumoniae* isolates. In clinical settings, we have identified various types of the *tmexCD-toprJ* gene cluster, among which two ST types (ST334 and ST39) carrying the *tmexCD2-toprJ2* gene cluster are reported for the first time. Additionally, a fusion plasmid containing both the *tmexCD-toprJ* gene and carbapenemase resistance gene, as well as a virulence gene, has been detected. Molecular epidemiological monitoring provides important evidence for bacterial resistance and evolution.

## Data Availability

The data supporting this study have been deposited in the National Center for Biotechnology Information (NCBI) database under BioProject ID PRJNA1394631.

## SUPPLEMENTAL MATERIAL

Fig. S1 spectrum.00689-26-s0001.docx Comparative genomic analysis of plasmids from three tmexCD-toprJ harboring isolates.

ASM does not own the copyrights to Supplemental Material that may be linked to, or accessed through, an article. The authors have granted ASM a non-exclusive, world-wide license to publish the Supplemental Material files. Please contact the corresponding author directly for reuse.
