# Supplementary material for: Characterization of tmexCD-toprJ-harboring plasmid in Klebsiella pneumoniae: insights into the molecular epidemiology of tigecycline resistance
Source: Microbiol Spectr. 2026 Aug 21;14(10):e00689-26. doi: 10.1128/spectrum.00689-26 (PMC13688829; doi:10.1128/spectrum.00689-26)
Supplement: Supplementary file 1 [file spectrum.00689-26-s0001.docx]

**Figure 1.** Comparative genomic analysis of plasmids from three *tmexCD-toprJ* [harboring isolates](https://www.ncbi.nlm.nih.gov/bioproject/1091228).The three circular plots, from the innermost to the outermost, represent GC Skew, GC content, and the aligned plasmid sequences, respectively. The outermost circle labels the names of genes and mobile elements. **A** pKP6_tmex(*tmexC2-tmexD2-TOprJ2*) and its aligned sequences *Klebsiella pneumoniae* pNUITM-VK2，*Klebsiella quasipneumoniae* pNUITM-VK4，*Klebsiella pneumoniae* pC6364_NDM and pKP14_tmex. **B** pKP14_tmex(*tmexC2-tmexD2-TOprJ2*) and its aligned sequences *Klebsiella pneumoniae* pNUITM-VK2, *Klebsiella quasipneumoniae* pNUITM-VK4 and *Raoultella ornithinolytica* p117885-OXY. **C** pKP21_tmex(*tmexC1-tmexD1-TOprJ1*) and its aligned sequences *Klebsiella oxytoca* strain p45-1, *Klebsiella pneumoniae* strain pT14441 and *Klebsiella pneumoniae* strain pTSYCC10-2 in the database.
